# The Association between Cafestol and Cardiovascular Diseases: A Comprehensive Review

**DOI:** 10.3390/medicina60060867

**Published:** 2024-05-26

**Authors:** Wen-Rui Hao, Chun-Yao Cheng, Huan-Yuan Chen, Jin-Jer Chen, Tzu-Hurng Cheng, Ju-Chi Liu

**Affiliations:** 1 Division of Cardiology, Department of Internal Medicine, Shuang Ho Hospital, Ministry of Health and Welfare, Taipei Medical University, New Taipei City 23561, Taiwan; b8501043@tmu.edu.tw; 2 Division of Cardiology, Department of Internal Medicine, School of Medicine, College of Medicine, Taipei Medical University, Taipei 11002, Taiwan; 3 Department of Medical Education, National Taiwan University Hospital, Taipei 100225, Taiwan; b05401143@ntu.edu.tw; 4 Institute of Biomedical Sciences, Academia Sinica, Taipei 11529, Taiwan; hchen9@ibms.sinica.edu.tw (H.-Y.C.); jc8510@yahoo.com (J.-J.C.); 5 Division of Cardiology, Department of Internal Medicine and Graduate Institute of Clinical Medical Science, China Medical University, Taichung City 115201, Taiwan; 6 Department of Biochemistry, School of Medicine, College of Medicine, China Medical University, Taichung City 404333, Taiwan

**Keywords:** cafestol, cardiovascular diseases, lipid metabolism, inflammation, endothelial function

## Abstract

Cafestol, a bioactive compound found in coffee, has attracted considerable attention due to its potential impact on cardiovascular health. This review aims to comprehensively explore the association between cafestol and cardiovascular diseases. We delve into the mechanisms through which cafestol influences lipid metabolism, inflammation, and endothelial function, all of which are pivotal in cardiovascular pathophysiology. Moreover, we meticulously analyze epidemiological studies and clinical trials to elucidate the relationship between cafestol and cardiovascular outcomes. Through a critical examination of existing literature, we aim to provide insights into the potential benefits and risks associated with cafestol concerning cardiovascular health.

## 1. Introduction

**Figure 1:**
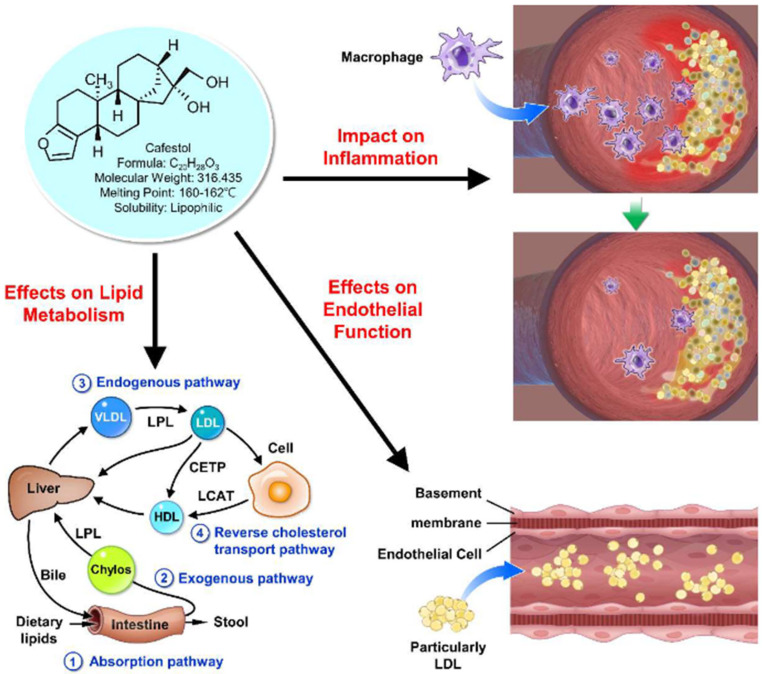
Cafestol’s promising effects on three pathological processes associated with cardiovascular diseases.

Coffee is indeed one of the most popular beverages worldwide, with an astonishing consumption rate of over 2.25 billion cups per day [1]. While its stimulating effects primarily stem from caffeine, coffee also contains a plethora of bioactive compounds that have sparked interest in their potential health implications. Among these compounds, cafestol, a diterpene found abundantly in coffee beans, has attracted particular attention for its potential impact on cardiovascular health [2]. Cafestol exhibits promising effects in mitigating various pathological processes associated with cardiovascular diseases (Figure 1). While numerous clinical studies have confirmed the association between coffee consumption and cardiac arrhythmia, the precise role of cafestol in the mechanism of cardiac arrhythmia remains unclear [3]. Studies have demonstrated that cafestol possesses anti-fibrotic properties, inhibiting high-glucose-induced cardiac fibrosis in cardiac fibroblasts and type 1-like diabetic rats [4,5]. This effect is attributed to the activation of nuclear factor erythroid-2 related factor 2 (Nrf2), a key regulator of antioxidant defense mechanisms, by cafestol [5]. Additionally, cafestol has been shown to inhibit urotensin II-induced cardiomyocyte hypertrophy, a common manifestation of cardiac remodeling, further highlighting its potential cardioprotective effects [5]. Moreover, cafestol exhibits anti-inflammatory properties by suppressing the expression of pro-inflammatory cytokines. It inhibits interleukin-8 (IL-8) expression in human umbilical vein endothelial cells (HUVECs) induced by urotensin II [6], as well as the production of interleukin-8 (IL-8), intercellular adhesion molecule-1 (ICAM-1), and monocyte chemoattractant protein-1 (MCP-1) in vascular endothelial cells stimulated by cyclic strain [7]. Thus, cafestol has been the subject of extensive scrutiny due to its diverse effects on key physiological processes such as lipid metabolism, inflammation, and endothelial function, all of which play crucial roles in the development and progression of cardiovascular diseases.

## 2. Mechanisms of Action

Cafestol exerts its influence on cardiovascular health through multifaceted mechanisms, impacting lipid metabolism, inflammation, and endothelial function. This section delves deeper into these mechanisms, elucidating the intricate interplay between cafestol and cardiovascular physiology.

### 2.1. Effect on Lipid Metabolism

One of the extensively studied mechanisms through which cafestol influences cardiovascular health is its effect on lipid metabolism [8]. Research indicates that cafestol affects lipid metabolism through various mechanisms. Cafestol has been found to increase fat oxidation and energy expenditure. Farias-Pereira et al. [9] demonstrated in a study using *Caenorhabditis elegans* that cafestol enhances fat oxidation and energy expenditure via a DAF-12-dependent pathway. Furthermore, cafestol has been identified as an agonist ligand for farnesoid and pregnane X receptors [10]. These receptors play crucial roles in regulating bile acid synthesis and cholesterol metabolism. Studies on animal models and humans have provided further insights into the impact of cafestol on lipid metabolism. Post et al. [11] showed that cafestol increases serum cholesterol levels in mice by suppressing bile acid synthesis. Additionally, in human studies, Urgert et al. [12] found that cafestol raises serum cholesterol levels and decreases serum lipoprotein(a) concentrations. Moreover, de Roos et al. [13] reported that cafestol increases plasma triacylglycerol levels by enhancing the production rate of large very-low-density lipoprotein (VLDL) apolipoprotein B. However, not all effects of cafestol are detrimental to lipid metabolism. Urgert et al. [14] found that cafestol decreases serum lipoprotein(a) levels in humans. Beynen et al. [15] also observed that boiled coffee, which contains cafestol, does not raise serum cholesterol levels in hamsters and rats. In addition to its effects on lipid metabolism, cafestol has been implicated in attenuating fibrosis in liver damage [16] and regulating cholesterol metabolism in human skin fibroblasts [17] and intestinal cells [18]. Thus, cafestol influences lipid metabolism through multiple pathways, including modulation of fat oxidation, bile acid synthesis, VLDL production, and lipoprotein concentrations. While it can increase serum cholesterol levels, it may also have beneficial effects, such as decreasing serum lipoprotein(a) concentrations and attenuating fibrosis. Further research is necessary to fully understand the net impact of cafestol on lipid metabolism and its overall health implications.

### 2.2. Impact on Inflammation

In addition to its effects on lipid metabolism, cafestol possesses anti-inflammatory properties that may contribute to its cardiovascular benefits. Several studies have investigated the anti-inflammatory effects of cafestol. Kim et al. [19] demonstrated that both cafestol and kahweol suppress cyclooxygenase-2 (COX-2) expression in macrophages, indicating their potential to modulate inflammatory responses. Ji et al. [20] reported that cafestol preconditioning attenuates apoptosis and autophagy during hepatic ischemia-reperfusion injury by inhibiting the ERK/PPARγ pathway. Lee and Jeong [21] found that cafestol protects against hydrogen peroxide-induced oxidative stress and DNA damage, suggesting its role in mitigating oxidative-inflammatory pathways. Moreover, cafestol has been shown to inhibit the production of pro-inflammatory mediators in vascular endothelial cells. Hao et al. [7] demonstrated that cafestol inhibits cyclic-strain-induced IL-8, ICAM-1, and MCP-1 production in vascular endothelial cells. These findings suggest a potential role for cafestol in mitigating vascular inflammation and endothelial dysfunction, which are key contributors to atherosclerosis and cardiovascular diseases. In addition to its effects on vascular inflammation, cafestol has implications for other inflammatory conditions. Islam et al. [22] provided insights into the therapeutic potential of cafestol in various health conditions, including its anti-inflammatory properties. Overall, the literature suggests that cafestol exhibits anti-inflammatory effects by modulating various inflammatory pathways, including COX-2 expression, oxidative stress, and cytokine production. These findings highlight the potential of cafestol as a therapeutic agent for inflammatory-related diseases, particularly cardiovascular diseases and liver injury.

### 2.3. Effects on Endothelial Function

Endothelial dysfunction is a hallmark of cardiovascular diseases and is characterized by impaired vasodilation, increased vascular permeability, and enhanced pro-thrombotic properties [23]. Cafestol has emerged as a promising modulator of endothelial function, with studies demonstrating its ability to improve vascular health and integrity. Studies have shown that cafestol possesses anti-inflammatory and anti-angiogenic properties, which contribute to its effects on endothelial function. Hao et al. [7] demonstrated that cafestol inhibits the production of pro-inflammatory cytokines, including IL-8, ICAM-1, and MCP-1, in vascular endothelial cells. This inhibition of inflammatory mediators suggests a protective role for cafestol in endothelial function. Furthermore, Wang et al. [24] and Moeenfard et al. [25] found that cafestol exhibits anti-angiogenic properties, which could potentially benefit endothelial function. Anti-angiogenesis refers to the inhibition of new blood vessel formation, a process crucial for maintaining endothelial integrity and preventing pathological angiogenesis associated with various cardiovascular diseases. Tsai et al. [6] also reported that cafestol inhibits urotensin II-induced IL-8 expression in HUVECs. Urotensin II is a vasoactive peptide implicated in endothelial dysfunction and cardiovascular diseases, and its inhibition by cafestol suggests a protective effect on endothelial function. Despite these beneficial effects, there may be complexities in the relationship between coffee consumption and endothelial function, as highlighted by Higashi [26]. The author discusses the “coffee paradox”, where coffee, despite containing potentially beneficial compounds like cafestol, has been associated with both positive and negative effects on endothelial function in different studies. Thus, cafestol exhibits anti-inflammatory and anti-angiogenic properties that may contribute to its beneficial effects on endothelial function. However, further research is needed to fully elucidate the mechanisms and potential implications of cafestol in maintaining cardiovascular health.

Overall, understanding the intricate interplay between cafestol and various aspects of cardiovascular health is essential for discerning its potential therapeutic or detrimental effects. The diverse pharmacological effects of cafestol underscore its potential therapeutic utility in preventing and managing cardiovascular diseases. By modulating these key physiological processes, cafestol may confer protective effects against cardiovascular diseases, including atherosclerosis, hypertension, and coronary artery disease. Further research is warranted to elucidate the precise molecular mechanisms underlying the cardiovascular effects of cafestol and to explore its potential therapeutic applications in cardiovascular disease prevention and management.

## 3. Epidemiological Evidence and Clinical Trials on Cafestol and Cardiovascular Health

Though the effects of cafestol alone on cardiovascular disease prevention and management are still unclear, epidemiological studies on coffee consumption and cardiovascular diseases reveal conflicting findings, indicating the complex relationship between coffee intake and heart health [27]. Methodological challenges, including variations in study design, population characteristics, and coffee consumption assessment methods, contribute to these inconsistencies. Notably, self-reported dietary surveys often introduce recall bias and misclassification, impacting study outcomes [28]. Thus, epidemiological evidence suggests a complex relationship between coffee consumption and cardiovascular disease (CVD). Bonita et al. conducted a comprehensive review, encompassing various study designs, including in vitro, cellular, animal, and human studies [29]. While some evidence suggested a potential protective effect of coffee against CVD, the overall findings were inconclusive due to the diverse effects of coffee’s composition on human physiology. The role of coffee brewing methods further complicates the association between coffee consumption and cardiovascular risk [30]. Different brewing techniques yield varying concentrations of bioactive compounds, notably cafestol [31]. For instance, espresso and French press coffee contain higher levels of cafestol compared to filtered coffee, potentially influencing cardiovascular outcomes [32]. While some studies suggest a higher risk associated with French-press coffee consumption [33], further research is warranted to elucidate the specific effects of cafestol across various brewing methods.

In clinical trials investigating cafestol supplementation’s effects on cardiovascular outcomes, mixed findings have been reported. Variations in study design, including differences in population characteristics, intervention duration, and outcome measures, contribute to these inconsistencies. While some trials suggest beneficial effects on lipid profiles and endothelial function with cafestol supplementation, others report no significant effects or even adverse outcomes (Table 1). Clinical trials have provided further insights into the effects of cafestol on cardiovascular health. Urgert et al. investigated the effects of cafestol and kahweol, two coffee diterpenes, on serum lipids and liver enzymes in humans [34]. Their findings revealed that both compounds significantly increased serum cholesterol levels, particularly LDL cholesterol, and liver enzyme levels, indicating a potential adverse effect on lipid metabolism and liver health. Subsequent studies by Urgert et al. further elucidated the individual contributions of cafestol and kahweol to elevated serum cholesterol levels [12]. De Roos et al. examined the absorption and urinary excretion of cafestol and kahweol in healthy volunteers with ileostomies, demonstrating significant bioavailability and systemic effects upon ingestion [33]. Studies by van Tol et al. [35] and van Rooij et al. [36] provided insights into the cholesterol-raising mechanism of cafestol, implicating increased serum lipid transfer protein activity. Additionally, genetic variability, as explored by Hofman et al., may modulate individual susceptibility to the effects of cafestol on lipid metabolism and cardiovascular health [37]. Furthermore, Grubben et al. investigated the impact of unfiltered coffee consumption on plasma homocysteine concentrations, revealing a potential contribution to cardiovascular risk [38]. These findings collectively underscore the multifaceted nature of the relationship between cafestol and cardiovascular health. The clinical significance of these findings remains uncertain, given the short duration of most trials and the lack of long-term data on cardiovascular outcomes. Further research, including larger-scale trials with longer follow-up periods and comprehensive outcome assessments, is necessary to better understand the effects of cafestol on cardiovascular health and inform evidence-based recommendations for cardiovascular disease management.

Overall, both epidemiological evidence and clinical trials highlight the complex interplay between cafestol and cardiovascular outcomes. Further research is warranted to elucidate the underlying mechanisms and develop strategies to mitigate potential risks associated with cafestol consumption while preserving the potential health benefits of coffee.

## 4. Potential Mechanistic Insights

**Figure 2:**
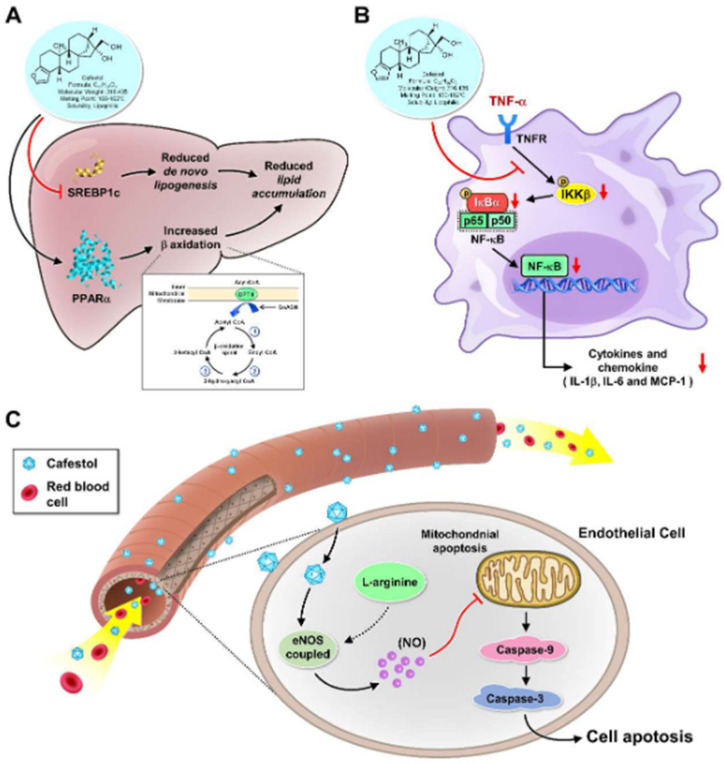
Proposed mechanisms between cafestol and cardiovascular diseases. (**A**) Cafestol modulates lipid metabolism via activating tsterol regulatory element-binding proteins (SREBPs) and peroxisome proliferator-activated receptors (PPARs). (**B**) Cafestol modulates inflammatory pathways that contribute to the pathogenesis of cardiovascular disease. (**C**) Cafestol exerts beneficial effects on endothelial function and provides potential cardioprotective effects.

Despite the divergent findings observed in epidemiological studies and clinical trials, preclinical research has offered valuable mechanistic insights into the association between cafestol and cardiovascular diseases (Figure 2). Animal studies, in particular, have provided a platform to elucidate the molecular mechanisms underlying the cardiovascular effects of cafestol, shedding light on its involvement in lipid metabolism, inflammation, and endothelial function. Understanding these mechanistic pathways could not only enhance our comprehension of the physiological effects of cafestol but also pave the way for the development of novel therapeutic strategies for managing cardiovascular diseases.

### 4.1. Role in Lipid Metabolism

One of the key mechanisms through which cafestol influences cardiovascular health is by modulating lipid metabolism. Animal studies have demonstrated that cafestol can affect various aspects of lipid metabolism, including cholesterol synthesis, bile acid metabolism, and lipoprotein metabolism. For example, research by Urgert et al. showed that cafestol supplementation in rodents resulted in a significant increase in serum cholesterol levels, particularly LDL cholesterol, by upregulating cholesterol synthesis and inhibiting bile acid synthesis [12]. These findings are consistent with observations from clinical trials, which have reported similar effects of cafestol on lipid profiles in humans. Moreover, animal studies have provided insights into the molecular mechanisms underlying the effects of cafestol on lipid metabolism. For instance, cafestol has been shown to activate transcription factors such as sterol regulatory element-binding proteins (SREBPs) and peroxisome proliferator-activated receptors (PPARs), which play key roles in regulating genes involved in cholesterol and lipid metabolism [8]. By modulating the expression of these genes, cafestol can influence cholesterol homeostasis and lipoprotein metabolism, ultimately impacting cardiovascular risk. Moreover, given its ability to modulate cholesterol metabolism, cafestol has garnered interest as a potential agent for managing dyslipidemia and cardiovascular disease. However, its cholesterol-raising effects may limit its clinical utility in individuals with pre-existing hypercholesterolemia or cardiovascular risk factors, necessitating further research to delineate its safety profile and optimal therapeutic application.

### 4.2. Role in Inflammation

In addition to its effects on lipid metabolism, cafestol has been implicated in modulating inflammatory pathways that contribute to the pathogenesis of cardiovascular diseases. Research suggests that cafestol may exert diverse effects on inflammatory processes through various molecular mechanisms. One significant mechanism through which cafestol impacts inflammation involves its modulation of cholesterol metabolism. Research by Post et al. revealed that cafestol increases serum cholesterol levels by inhibiting bile acid synthesis [11]. Dysregulated cholesterol metabolism is intricately linked to inflammation, with cholesterol metabolites acting as signaling molecules for pro-inflammatory pathways [10]. Thus, the cholesterol-modulating effects of cafestol may contribute to its influence on inflammatory processes. Moreover, cafestol exhibits anti-inflammatory properties in certain contexts. Arauz et al. demonstrated that coffee consumption mitigated fibrosis in a murine model of liver damage by downregulating the expression of pro-fibrotic mediators such as transforming growth factor-beta (TGF-β) and connective tissue growth factor (CTGF) [16]. By attenuating fibrosis, cafestol may alleviate inflammation associated with tissue injury and repair processes. In addition to its anti-fibrotic effects, cafestol has been implicated in cancer-related inflammation. Iwamoto et al. reported that cafestol, in conjunction with kahweol acetate, suppressed the proliferation and migration of prostate cancer cells [41]. Cancer-associated inflammation plays a pivotal role in tumor progression and metastasis, and the anti-cancer effects of cafestol may, in part, be attributed to its ability to modulate inflammatory signaling pathways within the tumor microenvironment [41]. Furthermore, cafestol interacts with xenobiotic metabolism pathways, potentially influencing inflammatory responses to environmental toxins. Huber et al. demonstrated that cafestol enhanced the activity of detoxification enzymes such as glutathione S-transferase (GST) and N-acetyltransferase (NAT), which play crucial roles in neutralizing genotoxic compounds [42]. By augmenting detoxification processes, cafestol may mitigate inflammation induced by exposure to environmental pollutants and carcinogens [42,43]. Moreover, cafestol’s anti-inflammatory properties make it an attractive candidate for conditions characterized by chronic inflammation, such as inflammatory bowel disease, rheumatoid arthritis, and metabolic syndrome [42,44]. Preclinical studies have demonstrated the efficacy of cafestol in ameliorating inflammation in various disease models [16,41], highlighting its potential as a therapeutic agent for inflammatory disorders. Hence, cafestol exhibits a complex interplay with inflammation, exerting both pro-inflammatory and anti-inflammatory effects depending on the context. Its modulation of cholesterol metabolism, direct interactions with inflammatory pathways, and enhancement of detoxification processes contribute to its therapeutic potential in managing inflammatory disorders. However, further clinical studies are warranted to elucidate the efficacy and safety of cafestol-based interventions in diverse patient populations. Overall, cafestol demonstrates a complex interplay with inflammation, exerting both pro-inflammatory and anti-inflammatory effects depending on the context. Further research is warranted to elucidate the precise molecular mechanisms underlying the dual role of cafestol in inflammation.

### 4.3. Role in Endothelial Function

Preclinical studies have suggested that cafestol may exert beneficial effects on endothelial function, thereby contributing to its potential cardioprotective effects. Hence, cafestol has garnered attention for its potential role in modulating endothelial function, which plays a crucial role in vascular health. Several mechanistic insights from in vitro studies shed light on the effects of cafestol on endothelial cells. Firstly, cafestol exhibits anti-angiogenic properties by inhibiting angiogenesis, the process of new blood vessel formation, which is essential for various physiological and pathological processes, including wound healing and tumor growth [24,25]. These findings suggest that cafestol may help regulate vascular remodeling by modulating angiogenic processes. Moreover, cafestol has been shown to mitigate endothelial dysfunction by suppressing inflammatory responses in endothelial cells. Studies have demonstrated that cafestol inhibits the expression of pro-inflammatory cytokines such as IL-8 and ICAM-1, which are implicated in endothelial dysfunction and atherosclerosis development [6,7]. Interestingly, despite its potential to improve endothelial function, the relationship between coffee consumption and endothelial health remains controversial, with some studies suggesting a paradoxical effect of coffee on endothelial function [26]. Further research is needed to elucidate the intricate mechanisms underlying the effects of cafestol on endothelial function and their implications for cardiovascular health. In terms of therapeutic potential, the anti-angiogenic and anti-inflammatory properties of cafestol make it a promising candidate for managing conditions associated with endothelial dysfunction, such as cardiovascular disease and diabetic vascular complications. By targeting key molecular pathways involved in endothelial dysfunction, cafestol may help preserve vascular integrity and function, thereby reducing the risk of cardiovascular events. However, it is essential to note that while in vitro and animal studies provide valuable insights into the potential therapeutic effects of cafestol, further clinical research is warranted to validate these findings in human subjects. Additionally, the optimal dosage and formulation of cafestol-based interventions need to be determined to ensure efficacy and safety in clinical settings. Thus, cafestol holds promise as a novel therapeutic agent for improving endothelial function and preventing cardiovascular disease by modulating angiogenesis and inflammatory responses in endothelial cells. Further investigation into the mechanistic insights and clinical implications of cafestol is crucial for harnessing its full therapeutic potential in vascular health.

### 4.4. Clinical Translation and Future Directions

The mechanistic insights gained from preclinical research on cafestol provide a foundation for translating these findings into clinical practice and guiding future clinical research. Understanding the molecular pathways through which cafestol influences lipid metabolism, inflammation, and endothelial function is crucial for identifying potential therapeutic targets and developing novel interventions for managing cardiovascular diseases. Future clinical studies should build upon the preclinical evidence and investigate the effects of cafestol supplementation on cardiovascular outcomes in human populations. Longitudinal studies with comprehensive outcome assessments, including measures of lipid profiles, inflammatory markers, endothelial function, and cardiovascular events, are needed to evaluate the long-term effects of cafestol on cardiovascular health. Moreover, randomized controlled trials are warranted to assess the efficacy and safety of cafestol supplementation as a potential adjunctive therapy for preventing or treating cardiovascular diseases.

In sum, the diverse pharmacological actions of cafestol have prompted investigations into its therapeutic potential. Preclinical research has provided valuable mechanistic insights into the association between cafestol and cardiovascular diseases, elucidating its roles in lipid metabolism, inflammation, and endothelial function. By understanding these molecular pathways, researchers can develop targeted interventions to mitigate cardiovascular risk and improve patient outcomes. However, the significance of cafestol lies in addressing a gap in research and the real world, particularly considering the limited studies in this area. Future clinical studies are needed to validate these findings in human populations and explore the therapeutic potential of cafestol in cardiovascular disease management.

## 5. Future Directions

As our understanding of the association between cafestol and cardiovascular diseases continues to evolve, several critical avenues warrant exploration to deepen our knowledge and guide future research efforts. Future investigations should aim to address key gaps in the existing literature, including elucidating the dose-response relationship between cafestol intake and cardiovascular risk, exploring the impact of different coffee brewing methods and types on health outcomes, conducting long-term prospective studies to assess sustained cafestol consumption, and conducting well-designed clinical trials to evaluate the therapeutic potential of cafestol in cardiovascular disease prevention and management.

### 5.1. Elucidating the Dose-Response Relationship

Understanding the dose-response relationship between cafestol intake and cardiovascular risk is crucial for informing evidence-based recommendations and public health guidelines regarding coffee consumption. Epidemiological studies should prioritize quantifying cafestol intake more accurately and investigating its specific effects on cardiovascular health across different levels of consumption. By assessing the dose-response relationship, researchers can determine the threshold at which cafestol intake may exert beneficial or detrimental effects on cardiovascular outcomes. Recent advances in dietary assessment methods [45], such as food frequency questionnaires and biomarker measurements, offer opportunities to improve the accuracy of cafestol intake estimation in epidemiological studies. Additionally, advances in analytical techniques, such as liquid chromatography-mass spectrometry, enable precise quantification of cafestol content in coffee and its metabolites in biological samples. These methodological improvements are essential for accurately assessing cafestol exposure and its association with cardiovascular diseases.

### 5.2. Impact of Coffee Brewing Methods and Types

The impact of different coffee brewing methods and types on health outcomes remains an area of active investigation [30,46]. Epidemiological studies should consider the diversity of coffee brewing methods, such as espresso, filtered coffee, and instant coffee, which yield varying concentrations of cafestol and other bioactive compounds. By examining the effects of different coffee types on cardiovascular risk, researchers can identify optimal coffee consumption patterns for cardiovascular health. Moreover, exploring the influence of coffee additives, such as milk, sugar, and flavorings, on the bioavailability and physiological effects of cafestol is essential for comprehensively understanding the health implications of coffee consumption. Animal studies and controlled human intervention trials can provide valuable insights into the mechanisms underlying the effects of coffee components on cardiovascular health and help elucidate potential interactions with other dietary factors.

### 5.3. Long-Term Prospective Studies

Long-term prospective studies are needed to assess the impact of sustained cafestol consumption on cardiovascular outcomes. While short-term clinical trials provide valuable insights into the acute effects of cafestol supplementation, long-term studies are essential for evaluating its chronic effects on cardiovascular risk over time. Prospective cohort studies with extended follow-up periods and repeated assessments of coffee consumption and cardiovascular outcomes are warranted to elucidate the long-term health implications of cafestol intake. Additionally, investigating potential effect modifiers and subgroups that may be particularly susceptible to the cardiovascular effects of cafestol, such as individuals with pre-existing cardiovascular risk factors or genetic predispositions, can help identify high-risk populations and inform targeted prevention strategies.

### 5.4. Well-Designed Clinical Trials

Well-designed clinical trials are essential for delineating the therapeutic potential of cafestol in cardiovascular disease prevention and management. Randomized controlled trials with rigorous methodology and appropriate control groups are needed to evaluate the efficacy and safety of cafestol supplementation in diverse populations. Trials should assess a range of cardiovascular outcomes, including lipid profiles, inflammatory markers, endothelial function, and cardiovascular events, to comprehensively evaluate the effects of cafestol on cardiovascular health. Moreover, exploring the potential synergistic effects of cafestol with other bioactive compounds in coffee, such as chlorogenic acids and trigonelline, may enhance its therapeutic efficacy and improve cardiovascular outcomes. Collaborative efforts between researchers, clinicians, and industry partners are essential for designing and implementing large-scale clinical trials that can provide robust evidence regarding the role of cafestol in cardiovascular disease prevention and management.

To put it briefly, future research efforts should focus on elucidating the dose-response relationship between cafestol intake and cardiovascular risk, exploring the impact of different coffee brewing methods and types on health outcomes, conducting long-term prospective studies to assess sustained cafestol consumption, and conducting well-designed clinical trials to evaluate the therapeutic potential of cafestol in cardiovascular disease prevention and management. By addressing these critical research gaps, we can advance our understanding of the association between cafestol and cardiovascular diseases and develop targeted interventions to mitigate cardiovascular risk and improve patient outcomes.

## 6. Conclusions

To come to the point, cafestol exhibits diverse effects on cardiovascular health through modulation of lipid metabolism, inflammation, and endothelial function. Although epidemiological studies and clinical trials have yielded conflicting results regarding the association between cafestol and cardiovascular outcomes, preclinical research suggests potential mechanistic insights. Future research endeavors should focus on elucidating the dose-response relationship between cafestol intake and cardiovascular risk, as well as assessing the long-term effects of cafestol on cardiovascular health. A comprehensive understanding of the association between cafestol and cardiovascular diseases is crucial for informing dietary recommendations and public health strategies aimed at reducing the burden of cardiovascular diseases.

## Figures and Tables

**Table 1:** Clinical trials of cafestol’s effects on lipid profiles are summarized in Table 1.

	Title	Authors	Years	Result
1.	Coffee consumption induces GSTP in plasma and protects lymphocytes against (+/-)-anti-benzo[a]pyrene-7,8-dihydrodiol-9,10-epoxide induced DNA-damage: results of controlled human intervention trials	Steinkellner et al. [39]	2005	Cholesterol slightly enhanced
2.	Consumption of French-press coffee raises cholesteryl ester transfer protein activity levels before LDL cholesterol in normolipidaemic subjects	De Roos et al. [32]	2000	Raise LDL cholesterol
3.	Diterpenes from coffee beans decrease serum levels of lipoprotein(a) in humans: results from four randomised controlled trials	Urgert et al. [13]	1997	Influence serum lipoprotein(a) levels.
4.	The cholesterol-raising diterpenes from coffee beans increase serum lipid transfer protein activity levels in humans	Van Tol et al. [34]	1997	Raise serum LDL and reduce HDL
5.	Separate effects of the coffee diterpenes cafestol and kahweol on serum lipids and liver aminotransferases	Urgert et al. [11]	1997	Raise total cholesterol, LDL, and fasting triacylglycerols; reduce HDL.
6.	Effects of cafestol and kahweol from coffee grounds on serum lipids and serum liver enzymes in humans	Urgert et al. [33]	1995	Raise serum cholesterol
7.	Diterpene composition of oils from Arabica and Robusta coffee beans and their effects on serum lipids in man	Mensink et al. [40]	1995	Elevated serum lipid levels
